# Men’s accounts of erectile function over time following radical prostatectomy: A narrative study

**DOI:** 10.1016/j.ijnsa.2026.100574

**Published:** 2026-06-02

**Authors:** Melissa Corbally, Catherine McGarvey, Carla O’ Neill, Barry Kestell

**Affiliations:** a School of Nursing and Midwifery, Trinity College Dublin, the University of Dublin, D02PN40, Ireland; b Mater Misericordiae Hospital, Eccles Street, Dublin, D07R2WY, Ireland; c School of Nursing, Midwifery and Health Systems, University College Dublin, D04V1W8, Ireland

**Keywords:** Erectile dysfunction, Nursing, Narrative research, Prostate cancer, Radical prostatectomy

## Abstract

**Background:**

Prostate cancer requiring radical prostatectomy surgery, invariably affects many aspects of men’s lives. Whilst altered sexual function is well documented as a potential outcome of surgery, the impact on men’s lives over time is little understood.

**Objective:**

This study explored men’s accounts of erectile dysfunction over three distinct time points: pre-operatively (n = 13), three months post operatively (n = 10) and six to nine months post operatively (n = 11).

**Design:**

Narrative Inquiry.

**Setting(s):**

A large tertiary teaching hospital in Dublin, Ireland.

**Participants:**

The first participant was recruited in August 2019. 18 men participated in a total of 34 interviews over three different time points.

**Methods:**

Narratives were examined individually initially then collectively to ascertain the elements of the unfolding story over time. Structural and thematic analysis comprised the analytic framework for the study.

**Results:**

Men’s accounts of erectile dysfunction were inextricably linked with masculine identity and subscription to masculine norms and accounts varied over time. Pre-operatively – men actively minimised erectile function over survivorship. This perspective changed in the two post-operative periods (most notably at 3 months) where men began voicing retrospective misunderstanding of the extent of their erectile dysfunction as well as regret and struggle with interventions aimed at optimisation of erectile function. Cost issues appeared to be challenging for men accessing care for penile rehabilitation. Throughout all narrative time points, age, fatherhood, relationship status and gender were identified as narrative rationalising strategies in their life stories including interaction/communications with healthcare professionals. Humour and metaphor assisted men’s expressions of this intimate, life altering experience.

**Conclusions:**

Men’s information and service needs relating to erectile dysfunction post radical prostatectomy vary over time. This highly masculinised experience of erectile dysfunction requires nurses to provide gender sensitive care pathways and survivorship care in a format which aligns both to men’s gender identity (and cost limitations) and is sequenced appropriately. Use of humour may be useful in assisting men’s engagement with information and care interventions. Clearer explanation of the impact of surgery pre-operatively including (clear distinction between erectile dysfunction and fatherhood at the point of diagnosis) as well as careful attention to men’s evolving understanding regarding erectile dysfunction would prove useful for effective survivorship.

**What is already known**

• Prostate cancer is the leading form of non-cutaneous male cancer worldwide and the second leading cause of cancer related deaths amongst men.
• Men undergoing radical prostatectomy surgery commonly experience erectile dysfunction.
• Little is known about men’s information needs and how men account for this experience over time.

Alt-text: Unlabelled box dummy alt text

**What this paper adds**

• This study found that men’s accounts of erectile dysfunction was inextricably linked with masculine identity and varied over time points.
• This study found that age, fatherhood, relationship status and gender were used by men to frame their narrative accounts at all time points.
• This study found that metaphor and humour were commonly used by men to articulate the experience of erectile dysfunction.
• Gender sensitive care pathways and survivorship care aligned to meet varying information needs have the potential to improve patient outcomes.

Alt-text: Unlabelled box dummy alt text

## 1 Background

Globally, prostate cancer remains the most common form of non-cutaneous male cancer representing the second leading cause of cancer related deaths amongst men (Sung et al., 2021). With the incidence of prostate cancer expected to rise in the next decade (Department of Health, 2022), the importance of the nursing response remains paramount. In Ireland, approximately 28% of all men undergo radical prostatectomy as part of their treatment strategy. The decision to operate is a considered one, taken following careful discussion with the man regarding the rationale, expected benefits and alternative options. The procedure is complex and considers patient as well as clinico-pathological factors. Radical prostatectomy can be performed using robotic, open or laparoscopic techniques with nerve or non-nerve sparing options depending on the case. Men experience a variety of care needs and sequelae from this experience including erectile dysfunction. This brings particularised challenges for nurses in providing timely and sensitive care.

It has been well acknowledged in the literature that prostate cancer and its subsequent treatment can have a significant impact on men’s masculine identity (Corbally et al., 2023; Kong et al., 2017; Chambers et al. 2017,). Masculinity refers to ‘men’s identity or how they make sense of themselves as a man’ and it also frames how men make sense of what is happening to them. Masculinity is not static, rather it is fluid and subject to change (Connell and Messerschmidt, 2005). Men’s masculine identity is disrupted by the embodied experience of prostate cancer and radical prostatectomy, this disruption may be temporary or permanent and indeed the men themselves do not know what the long-term disruption will be. Intimate citizenship (Plummer, 2003) is a concept which recognises how individuals navigate intimate and personal aspects of their life within public spheres. This perspective has clear resonance for men experiencing erectile dysfunction as this intimate function through interactions with nurses, doctors, partners and other conversations emerges into a public entity not by choice, but by necessity. There is a notable dearth of evidence in relation to men’s experiences and accounts of erectile dysfunction, particularly over time. This study sought to generate new information about how men’s needs evolve over time.

## 2 Methods

### 2.1 Aims and objectives of study

The primary aim of the study was to explore men’s life stories of radical prostatectomy. The objectives of the study were to examine how a cancer diagnosis and radical prostatectomy impacts on a man’s life, to explore narrative strategies and language men use in talking about their prostate cancer experience, to identify areas of key concern to men’s identity and survivorship potential, to explore the sense, men with prostate cancer make of aspects of the current care pathways and to explore men’s information needs at particularised points in the cancer/recovery journey

Men’s broad sense making processes relating to a cancer diagnosis have been previously published (Corbally et al., 2023). This paper presents findings specifically relating to erectile dysfunction experienced by men and subsequent information and service needs particularised to each point in the recovery journey. Ethical approval for the study was granted through the institutional review board of the study hospital (ref: 1/378/2060).

### 2.2 Study design

This study used narrative inquiry, a qualitative approach to investigate participant life stories. Because a cancer diagnosis impacts on one’s biography and identity, narrative inquiry was specifically chosen due to its scope in exploring lived lives, told stories, particularised language and meaning whilst appreciating multiple contexts, voiced knowledge and the influence of time and key turning points (Riessman, 2008).

### 2.3 Study setting and participants

The study setting was a large tertiary hospital in the Republic of Ireland. Participants were recruited from general urology, rapid access and pre-operative assessment clinics. Informed consent was obtained from all participants prior to data collection. 18 men participated in a total of 34 interviews over three different time points: pre-operatively (n = 13), three months post operatively (n = 10) and six to nine months post operatively (n = 11).

### 2.4 Data collection

All interviews were carried out on site. Data was collected by the project researcher and consisted of an open-ended biographical interviewing technique whereby participants responded to a single open ended narrative framing question to elicit men to talk about all the events and experiences that were important to them at that particular point in their life story (Wengraf, 2001). Following this single question, clarifying questions were asked but could only relate to the original story told by the participant. The narrative framing question was as follows:

“As you know, I am researching men’s stories of the experience of prostate cancer and its treatments. I understand you have had this experience. So please can you tell me your story, all the events and experiences that were important for you up until now. You can start wherever you like. I’ll listen first and won’t interrupt. I’ll just take some notes and might ask you some follow up questions afterwards”.

This single narrative question was asked at every time point and proved very effective in eliciting high quality narrative data. Interviews lasted between 40 and 60 min, were recorded and transcribed verbatim.

### 2.5 Data analysis

With the assistance of Nvivo Version 13, transcribed data was analysed according to Riessman’s thematic and structural narrative technique (Riessman, 2008) in conjunction with the research objectives. The project researcher and principal investigator performed initial coding and regular research team meetings facilitated upholding methodological rigour and the evolving analytic process of reflexive cross-comparison with emergent thematic and structural findings presented below.

## 3 Results

### 3.1 Characteristics of participants

Pseudonyms were used to protect the participants’ identity. John, James, Nathan, Paul, Gavin, Evan and Nicholas were interviewed at all three time points. Alexander was interviewed at two time points with Roger, Charles, William, Henry, Colin, Edward, Nigel, Glen, Simon and Joseph being interviewed at one time point. Table 1 below provides an overview of the participants and the time points in which they were interviewed.

**Table 1:** Overview of participant group.

Pseudonym	age	Gender	Pre op ED	Ethnicity	Method	Gleason grade	Nerve Spare	Final histology	Interview 1	Interview 2	Interview 3	In relationship	Previous treatment
John	64	AMAB^1^	N	African	Rob	3+4	Non nerve spare	Grade group 2 pT3a	X	X	X	Y	AS
Nathan	56	AMAB	N	white Irish	Rob	4+3	Bilateral nerve spare	Grade Group 2 3+4 pT2	X	X	X	Y	RXT opinion
Gavin	49	AMAB	N	white Irish	Rob	3+4	Unilateral right nerve spare	Grade group 2 3+4 pT2c	X	X	X	Y	AS
Evan	62	AMAB	Y	white Irish	Open	4+4	Non nerve spare	Grade group 3 pT3b 4+3	X	X	X	Y	RXT opinion declined
Nicholas	66	AMAB	N	white Irish	Open	3+4	Non nerve spare	Grade group 2 pT2c	X	X	X	Y	AS, RXT opinion
Paul	52	AMAB	y	white Irish	Rob	4+3	Bilateral nerve spare	Grade group 3 4+3 pT2nx	X	X	X	Y	RXT opinion
James	58	AMAB	N	white Irish	Rob	3+4	Non nerve spare	Grade group 3 4+3 pT2nx	X	X	X	N	rXT not an option UR
Alexander	67	AMAB	Y	African	Rob	3+4	Non nerve spare	Grade group 2 pT2c	X	X		Y	No
Henry	48	AMAB	N	white Irish	Rob	3+4	Bilateral nerve spare	Grade group 2 pT2c	X		X	N	No
Glen	60	AMAB	N	white Irish	Rob	3+3	Non nerve spare	Grade group 2 pT2c	X			Y	No
Charles	52	AMAB	N	white Irish	Rob	3+4	Nerve sparing procedure	Grade group 2 pT2c	X			Y	No
William	72	AMAB	N	white Irish	Rob	3+4	Non nerve sparing	Grade group 2 pT2	X			Y	DXT opinion
Joseph	67	AMAB	N	white Irish	Rob	4+3	Non nerve sparing	Grade group 3 PT3a 4+3	X			Y	AS
Roger	69	AMAB	N	white Irish	Open	3+4	Non nerve sparing	Grade group 2 pT3a		X		N	No
Simon	71	AMAB	N	white Irish	Open	4+3	Non nerve sparing	Grade group 2 pT3a 3+4		X		Y	DXT opinion
Nigel	48	AMAB	N	white Irish	Open	3+4	Unilateral nerve spare difficult	Grade group 1 pT2a			X	N	No
Colin	69	AMAB	Y	white Irish	Open	3+4	Non nerve sparing	Grade group 2 pT3a			X	Y	DXT opinion
Edward	52	AMAB	N	African	Open	4+3	unilateral nerve spare	Grade group 3 pT2a			X		DXT opinion

1 Assigned Male at Birth

### 3.2 Overarching narrative themes

Masculine identity positioning, metaphor and humour were utilised in assisting the conveying of meaning throughout all narrative time points.

In framing their life stories, the inextricable link between masculinity, subscription to masculine norms, erectile function and sex was clearly evident and loss of this was identified by some as undermining masculinity. It is interesting that men used women to emphasise their gendered plight.

‘…you know obviously sex is very important for males and females you know clearly with males losing that function, it's like losing the manhood’ (Paul, Pre-operatively)

‘I suppose it’s like when a woman gets a hysterectomy or gets her breasts removed…the woman would probably worry that you know she’s not as feminine…you hear them saying it's unattractive…I'll be honest with you I’m feeling the same….’ (Charles, Pre-operatively)

Aspects central to masculinity (age, fatherhood, relationships and gender) were used by men to frame their stories relating to erectile dysfunction throughout their narratives. Men used humour and metaphor in their narrative expressions of this challenging experience. Use of metaphor is demonstrated in the following example:

‘I was heading towards the age of probably hanging up the gun anyway (laughing) so eh, so to speak you know. So the gun was going to be going up” (Evan, 9 months Post-operatively).

In particular, humour was made use of by the men when discussing penile rehabilitation experiences

‘[Doctor] says you know we have a pump clinic every four to six weeks. I said really? He said but you have to bring your own. What no sharing?’ (James, 3 months Post-operatively).

The metaphor of an engine was used to explain erectile functionality

‘Em it's just I suppose it's almost like you don't function properly. It's not that you'd feel any less you wouldn't feel any less a man if that makes sense….it’s like an engine in the car you know it drives but you know there's a niggly couple of little problems with the engine…it's not a fully functioning engine it's not at 100% capacity.’ (Gavin, 9 months Post-operatively).

‘Age’ entered into the narratives of the men in a number of ways, taking into account their experience of erectile dysfunction, their expectations for recovery or explaining current progress with recovery. For some men, age played an important role in their interpretation of their loss of erectile function, with most men, across each time point, suggesting that sexual dysfunction would be more challenging for younger men than older men. Being in a later stage of life was imagined to make it easier to accept issues with erectile function.

‘…the main focus now….comes back to the whole phase three part of the recovery the erectile dysfunction…it's one of these things where by because you're young compared to other men who would have it. I suppose erectile dysfunction and the whole sexual dysfunction thing for men of a certain age becomes irrelevant because they've reached a point in life where it's not important to them but when you're younger, fourties, fifties, it is still important and you feel that you've lost or you've potentially lost out on your or I suppose your function as a man. (Gavin, 9 months Post-operatively)

Humour appeared to be used by several of the men when discussing sensitive topics, the example below relating to use of a pump during sex.

‘I go upstairs, I got the pump, ‘hold on love’ (makes pumping motion)…you know, you laugh about it. If you can't laugh about stuff you’d cry’.’ (Evan, 3 months Post-operatively).

In addition to the overarching themes, men utilised narrative framing strategies relating to the experience of erectile dysfunction particular to each time period. These are presented further below.

### 3.3 Pre-operative accounts of erectile dysfunction: ‘Sex versus do you live’

Pre-operatively, mortality was the men’s overriding concern. Participants prioritised ‘living’ over ‘living well’ and erectile function was effectively minimised. Men’s accounts tended to be pragmatic. “There's a weighing scales you know, 'sex' versus 'do you live'. There's no, there's no choice on that…’ (Evan, Pre-operatively)

#### 3.3.1 ‘Fathering a child’

Although being a father and age were used as rationalising techniques, it is of note that men stated that ‘fathering a child’ was a means by which healthcare providers communicated potential post operative erectile dysfunction. Although this statement implicitly includes sexual function and reproductive function, the men seemed to merge both suggesting that because they had children, they had no real concern about sexual function.

‘…[surgeon] discuss (sic) with me the side effects…if I choose to remove it he said I will not be able to father a child. So, I’m good because I am 62 years old so…I’ve got two daughters so….’(John, Pre-operatively).

#### 3.3.2 Relationships and age

Pre-operatively, it seemed that men expressed age and relationship status together. The value men placed on loss of a sexual relationship seemed to vary. For some it was a big issue, for others is was not.

‘[Doctor said] 'you won't be able to have any more children'. That's no problem at my age, you know, 62. And the chance for the erection goes…I'm still sexually active…..at the age. There's loads of friends of mine that wouldn't have had sex the last 10 years. Just we've been very happy” (Evan, Pre-operatively)

By contrast Nathan suggested that the loss of erectile function would not make much difference to him.

‘My wife and I don't really have much of sex life and that's really down to me. And it's a source of eh it's a source of disappointment and frustration and anger with my wife…..I'm not going to be losing an awful lot if you follow me? I'm 56, I've four children. (Nathan, Pre-operatively)

Paul expressed a concern about erectile function due to the effect it may have on him and his partner.

‘You know from my point of view it's one thing but from my wife's point of view it's another. And in fairness to [Doctor] he looked me straight in the eyes and said ‘well done you because not many people think of the partner’ (Paul, Pre-operatively)

One man who was not in a relationship suggested that sexual function was not a concern for him at the moment as it was not something that featured in his life.

#### 3.3.3 Challenges with information

Although the men received information from healthcare staff regarding erectile dysfunction pre-operatively, their ability to process and absorb this information was challenging.

“I don't think [Surgeon] told me a whole lot about the surgery. I suppose I got I got a wee leaflet, a wee bit of information. The nurse…she put a heap of stuff by me the last day and it's difficult to take, you can only take bits of it in” (Glen, Pre-operatively)

“I’ve got loads of information and loads of booklets. I’ll never read them all. I hate reading but I’ll skip through them. (Charles, Pre-operatively)

In summary, men’s accounts were markedly determined by a key focus on their mortality where they actively minimised erectile function and there were clear deficits in receiving, understanding and/or interpreting information. This experience evolved with time with a marked shift as illustrated below.

### 3.4 Three months post operatively – misunderstanding the cost (psychological and financial) of erectile dysfunction

This time period seemed to be the most crucial and devastating for participants and was characterised with extensive retrospective misunderstanding of the psychological and financial costs of erectile dysfunction. Erectile dysfunction was evident in all men’s narratives at three months post-surgery. This time point illustrated the full impact of the diagnosis and effects of the surgery.

‘The consequences, I'd say I probably didn't take it all in you know first-hand really especially the sexual end of that I didn't really pass much heed of that really it's only when you're out and having it done and you look back’ (Nicholas, 3 months Post-operatively).

In addition to this lack of understanding, participants believed that doctors failed to emphasise important information inhibiting nurses’ desire to explain the full implications of erectile dysfunction leading to disappointment.

‘…I think the sexual end of it should be explained more you know that it's not there's no going back to normal like you know….that sort of thing should be emphasised….I think they do fluff that, the doctors more so I think. The nurses wanted to explain it but I think the doctors just kind of want to say you know you'll get Viagra and things. You form the opinion ‘Ah sure I'll be back in business in two months’ (Nicholas, 3 months Post-operatively).

On the contrary, Nathan was aware of the long process of penile rehabilitation.

‘My understanding is that …in many cases it does come back but I think it's more likely to be in 9 months to 18 months or even two years’ (Nathan, 3 months Post-operatively)

Participants continued in narrating the varying solutions they were attempting to resolve their erectile dysfunction and in doing so, unearthed further misunderstanding, reservations and disappointment. Men expressed reservations about using erectile dysfunction aids. Alexander, for example, had a bad reaction to Sildenafil previously and was reluctant to do so now.

‘When I take the Viagra (Sildenafil) it makes me, I see different colour lights…I feel nauseous just so I don't I don't take any of those anymore.’ (Alexander, 3 months Post-operatively)

Several men were seeing some response at this point, with Evan achieving erections with the assistance of the vacuum pump.

‘…I'm using the pump for three weeks now…I haven't tried it for sex they said in about a month if you want. I don't know whether I would.’ (Evan, 3 months Post-operatively)

Paul was achieving erections capable of sexual intercourse, with the use of Tadalafil.

‘The sexual maybe 80 or 90% and I'm in a very good place mentally.’ (Paul, 3 months Post-operatively)

However, Paul’s success with sexual function brought the new challenge of ‘climacturia’, which he didn’t expect to experience but was attempting to manage.

‘When I climax now I don't, I don't ejaculate…a bit of urine does come out. Em so that was a little bit of a surprise’ (Paul, 3 months Post-operatively)

James reported that he was not interested in restoring erectile function and as a result believed it was difficult to justify the expense of the erectile function pump in his discussions with the surgeon. He used humour to augment his position.

‘[Surgeon] talked about pumps and I was like no. The Cialis (Tadalafil) thing I can do once it doesn't cost a fortune because there's no point in spending the money…He says you know we have a pump clinic every four to six weeks. I said really? he said but you have to bring your own. what no sharing? so I'd have to invest a couple of hundred quid to come and play with it here with other people.’ (James, 3 months Post-operatively)

Several men expressed surprise at the costing of ED rehabilitation as articulated by Evan who was prescribed a PD5 inhibitor (not subsidised by the national drug payment scheme) by the doctor.

‘The only thing I said about the medication…I’m not a medical card [holder]. And I said those tablets are five euros each, and he said ‘oh I didn't think they were that dear’. So he said to me ‘I might change you onto Viagra (Sildenafil). Viagra will do the same thing but it's they've come down in price’ (Evan, 3 months Post-operatively)

Whilst most men had begun some treatment for restoration of erectile function at the 3 month point. It was clear that men were compromising on care due to the burden of the cost of such care. Gavin recognises that this burden extended to his family also.

‘It's very expensive…it's not recognized, it's not coded for the drug payment scheme… It is permanent 134 Euros per month for the medicine… On the practical level you'd have to consider the benefits of whether it would be any good” (Gavin, 3 months Post-operatively)

#### 3.4.1 Impact on sexual relationships: loss of spontaneity

Another ‘cost’ for men at this time point who had some success with treatment for erectile function was a loss of spontaneity which necessitated planning for sexual intercourse.

‘You need to time these tablets you know a little bit. It you know as my wife said to me she ‘How long will it take?’. ‘Well they say about 1/2 an hour. Well sure I'm better giving it an hour.’ So you know, it's not as spontaneous as it used to be…’ (Paul, 3 months Post-operatively).

This was experienced as anxiety provoking for Paul.

‘….You're thinking during the whole thing will the tablet work? …thinking about it too much can ruin the moment so to speak.’ (Paul, 3 months Post-operatively).

The psychological impact is demonstrated by John.

‘If I try and if I failed it will put more pressure on me, on my head…if I try and it's not positive you know it's going to add to my problem.’ (John, 3 months Post-operatively).

Evan used humour to explain lack of spontaneity

‘ I was having a joke…saying you know I said to my wife hold on about 5 min I've just to go upstairs I'll give you the whistle or I'll bang the roof to come up when I have this thing sorted out. Whereas, whereas sex is generally spontaneous….’ (Evan, 3 months Post-operatively)

The importance of strong relationships and creativity was emphasised by some participants as helpful..

‘I'm just happy quite happy the way I am. I think to be honest with you when you reach a certain age….you can still love your missus….and you can still have a cuddle and still have a real laugh’ (Simon, 3 months Post-operatively).

‘There’s plenty more ways you can be involved with somebody sexually’ (James, 3 months Post-operatively).

Whilst the realisation of the ‘cost’ of erectile dysfunction began at 3 months, the ongoing impact of this experience endured and is discussed further below.

### 3.5 Six to nine months post operatively- ongoing challenges

At six to nine months post-surgery erectile dysfunction remained problematic for most men. Several of the men stated that they saw little progress from three months to nine months with little apparent understanding of the process of rehabilitation. Evan, for example, was still making limited use of the pump.

‘If you were able to have an erection from the pump then there was no need for the Viagra (Sildenafil) he said you know…I haven't used, I haven't tried it for anything else for sex or anything like that….’ (Evan, 9 months Post-operatively)

Gavin, was still struggling.

‘ There is none there is none at all. No, you wouldn't you just wouldn't get an erection at all that's basically it…I was told that part of the recovery could take anywhere up to two years which is fair enough. (Gavin, 9 months Post-operatively)

Nigel similarly, had not been able to achieve an erection by nine months, prioritising his urinary function and minimising ED.

‘I haven't had any sort of reaction…it's not a big deal'. If I can get the bladder dysfunction sorted that would be for me… my main priority at the moment you know’ (Nigel, 9 months Post-operatively)

The influence of other men on participants was also evident.

‘The erection thing, that hasn't happened or even looked like happening. That's that end of it so…a couple of the guys that I had a meeting with up in the [hospital] they didn't do it either you know what I mean? They didn't feel that it was suitable for them and that, so I didn't go down that road’

(Nicholas, 9 months Post-operatively)

Henry had successfully achieved erections capable of sustaining sexual intercourse. In both cases this was achieved with the use of medication and without the use of the vacuum pump.

‘…you know I actually haven't used the pump for we'll say for an erection we just for the rehabilitation more than anything else yeah…taking the tablets…yes it has worked.’ (Henry, 9 months Post-operatively)

The traditional male role of men initiating sex was highlighted by Evan who in spite of a lack of dialogue regarding sex, problematic nerve sparing and impacted intimacy, a strong relationship is possible.

‘It hasn't changed our relationship in regards to one another…the sexual part hasn't come up at all…in probably a lot of relationships …it would be more where the male is the instigator…Since the operation we haven't really spoken about the sex part….it just hasn't come up…And I had told her I was taking the Viagra (Sildenafil) and they had to take away more of the nerve ending than they had initially thought because it had gone slightly in the back and that there was nothing happening there so. So she just said ‘fine’ you know so. It hasn't changed our relationship in regards to one another you know so …..intimacy sort of sort of went out the window (Evan, 9 months Post-operatively)

Gavin, by contrast, spoke openly with his wife about his erectile dysfunction and expressed how her understanding and encouragement helped reinforce their relationship.

‘She would have a very pragmatic approach to things… ‘look it's another phase in your recovery…but you have to remember that your body went through a traumatic event and you're the nerves some were saved others weren't so it's going to take time for your body to physically repair itself so don't beat yourself up’ She said ‘it doesn't affect our relationship whatsoever’. (Gavin, 9 months Post-operatively)

## 4 Discussion

The Global Better Sex study undertaken across 27 countries internationally (N = 12,653) demonstrates an inextricable link between erection hardness, satisfaction with sex life, intimate relationships and overall health (Mulhall et al., 2008). Men undergoing radical prostatectomy by virtue of surgical intervention risk losing facets of their health through their guided ‘choice’ to undergo surgery. The fundamental importance of expressing sexuality and its connection to wellbeing is an area central to nursing as well as many frameworks and models of practice mandating nurses to address this aspect as part of holistic nursing care delivery (Holland and Jenkins, 2019). However, lack of preparedness amongst nurses and healthcare providers has been consistently identified as an inhibiting factor (Neenan and Chatizi, 2025; Walker et al., 2021). The expression of sexuality is a central tenet of nursing practice, and the fact that men experience a multiplicity of sexual conditions and unmet needs associated with radical prostatectomy experience over time suggest that the need for evidence-based nursing interventions in prostate cancer care is urgent. The relatively new development of the field of oncosexology (Salter et al., 2021) is testament to the multidisciplinary concerns regarding unmet needs.

Masculinity is often closely tied to control of the body and sexual prowess (Connell, 2005), both of which are undermined, for a period at least, by radical prostatectomy surgery. Whilst the inextricable link between erectile dysfunction and masculinity has been asserted (Chambers et al., 2017), this study provides new understandings regarding how this masculinity (through the radical prostatectomy and erectile dysfunction experience) evolves over time, highlighting a need for an evolving and tailored nursing response. This study illustrates how the evolving masculinized experience is framed by men’s subscription to masculine norms (which can be beneficial or inhibiting) as well as being shaped by humour & metaphor. The evolution of men’s stories from being initially survival based to loss-based presents findings which demonstrate evolving deficits and potential areas for effective, gender sensitive nursing intervention. Pre-operatively, men’s pre-occupation with life over life quality is understandable and is reflective of normal human coping mechanisms in preserving life at all costs. Healthcare providers seemed to discuss sexual function through ‘having more children’ with erectile function implied indicating potential for misunderstandings between healthcare provider and patient. In this study, the stark contrast of retrospective misunderstanding of what they were ‘told’ by healthcare providers points to a real opportunity for nurses and healthcare providers to consider reinforcement of information and care at this crucial time point to minimize adverse consequences.

We suggest that findings from this study can be incorporated into nursing practice through use of the PLISSIT model. The PLISSIT or P-LI-SS-IT model devised by Annon (1976) is a sequentially advancing method of care used to assist care givers in framing conversations in relation to sexual health. PLISSIT is a holistic and adaptable approach which accompanies clinical history (findings from this study would suggest nurses need to also provide time for men to tell their story, notice how men make sense of the experience over time, recognize issues of importance to them and respond accordingly). The adaptability and flexibility of the tool relates both to patients and healthcare providers who have varying experience in this area, allowing for a range of treatment choices to be offered (Annon, 1976).

The terms in the PLISSIT acronym stand for; Permission, Limited Information, Specific Suggestions and Intensive Therapy and has been found to be effective in promoting self-efficacy amongst men (Abed et al., 2022). Developed by Annon (1976), Permission relates to creating space to discuss thoughts, feelings and behaviours. This study has identified that not all men were afforded this opportunity. Further, in instances where permission was given, there is a need for such permission to be afforded to the client at particularized intervals to allow for evolving experiences. It is of note that Permission is potentially bi-directional reflecting staff skill challenges, personal challenges and lack of time identified in studies of factors inhibiting nurse-led sexual support conversations (Bingham et al., 2022; Neenan and Chatzi, 2025).

Self-report pre-assessment questionnaires (e.g. the International Index of Erectile Function (IIEF) (Rosen et al., 1997)) enable men to provide information in a more naturalistic environment. The five factors identified by the IIEF (erectile function, orgasmic function, sexual desire, intercourse satisfaction and overall satisfaction) show high specificity and sensitivity. The Expanded Prostate Cancer Index Composite (EPIC) (Wei et al., 2000) may also be worth considering in prostate cancer patients experiencing urinary, bowel, sexual and hormonal challenges. However, both tools are limited by their mechanistic focus and do not account for the wider biopsychosocial impacts and masculinized expressions as identified in this study. Tools such as these (with modifications) offer real potential for nurses to initiate productive therapeutic conversations.

Limited Information relates to clinicians providing specific and targeted information (Annon, 1976). This study identified that information provided although specific, was insufficient to incorporate attending to men’s identity positioning, narrative framing and potential for retrospective regret when designing nursing interventions. Although a prostate cancer diagnosis can come as a shock (Corbally et al., 2023), tailoring the information with staged communication intervals may prove useful.

Making Specific Suggestions the third phase, requires that nurses a priori ascertain the perspective and understanding of the patient in addition to deepening clinical assessment (Annon, 1976). This is a unique opportunity for nurses to understand narrative structures which serve an important communicative function in conveying meaning (Bruner, 2004; Riessmann, 2008). In this study, masculinity, age, fatherhood, relationships and gender were used by men to position themselves throughout the life story. Nurses, who interface with men experiencing this potentially life changing surgery need to appreciate how men make sense of these challenging experiences, as well as recognizing time sensitive aspects. Shame, embarrassment and lack of confidence were identified by Neenan and Chatzi (2025) as key emotions inhibiting nurses in discussing sexuality related care in interventions. Whilst humour and metaphor could be interpreted as not taking one’s health seriously, this study identified that for men, this method proved to be an important narrative tool to aid the discussion of life altering experiences. Pragmatic suggestions regarding penile rehabilitation are potentially possible also at this point.

Intensive Therapy, the fourth phase relates to expert provision of extensive therapeutic support (Annon, 1976). This perhaps is best reserved for Advanced Nurse Practitioners, Clinical Nurse Specialists (or their equivalent) or Consultant led care provision. However, the first three phases of PLISSIT provides substantial structure for all other nurses and healthcare to improve men’s quality of experience going forward. The potential for nurse-led telehealth at targeted time periods (initiated in the study hospital as a result of findings) also offers real potential in improving accessible and timely support for men (Kwok et al., 2022).

Age was used as a narrative positioning tool by men in this study to account for their choices. This phenomenon, previously identified (Kong et al., 2017). highlights how men’s initial concern with life over quality of life allowed them to deal with this disruption in the short term. Ironically, age also frames nurses’ perceived willingness to initiate/inhibit ED discussions (with advanced age and clinical experience associated with promoting discussions) (Neenan and Chatzi, 2025). Fatherhood and relationships with partners were also notable narrative framing strategies pre-operatively. Whilst this aided men to justify decision making at this point, future nursing care interventions and discussions would benefit from using similar narrative strategies to assist men’s understanding of the full consequences of their decision making.

Permission for nurses and radical prostatectomy patients to explore these limiting assumptions would be helpful.

It was evident that some of the men used humour and metaphor to articulate the challenges they were experiencing as a result of erectile dysfunction. This has been acknowledged as a mechanism that men utilise to maintain stereotypical masculine norms of stoicism and emotional control in the face of adversity (Gannon et al., 2010). Additionally, the men described being overwhelmed by information that was provided suggesting that the content, form and absorption of healthcare information about the consequences of erectile dysfunction requires judicious consideration. Meeting information needs is a fundamental practice of nursing. This study highlights the importance of healthcare providers appreciating men’s communication practices which include humour and metaphor to ensure information needs are met in a sequenced, appropriate and understandable format. Use of practical metaphors such as engines to explain function for men assists a masculine focus on ‘pragmatic embodiment’ which means maintaining a functioning body that allows them to fulfill their gender specific role (Watson, 2000, p.68). This may be helpful in developing information resources with Specific Suggestions worded using men’s own language to foster a sense of engagement.

The three month post operative point was a crucial turning point in the experience of erectile dysfunction as it illustrated men’s accounts of the ‘costs’ and subsequent losses at several levels and identifies a real need for healthcare intervention at this timepoint as the embodied experience of erectile dysfunction was significant for most men in this study as not only had they lost functionality of a part of the body, they had lost their ‘manhood’. Penetrative sex was constructed as central to masculine identity (Gannon et al., 2010; Walsh and Hegarty, 2010) and although some accepted erectile dysfunction, loss of erectile function is portrayed as ‘not fully functioning’ as a man. Once the men had overcome the threat to their mortality the impact and reality of radical prostatectomy and other associated losses and costs become more apparent. In other cases, men privileged pleasing one’s partner sexually over being able to engage in penetrative sexual intercourse with the effect of reconstructing masculinity in a way that falls in line with their current capacities (Oliffe, 2005). Whilst this was in contradiction to large scale research (e.g. Mulhall et al., 2008), perhaps this minimisation is understandable.

The financial, psychological and emotional cost of erectile dysfunction through the embarrassment of penile rehabilitation, the unaffordability of penile pumps and medications, ongoing flaccidity or lack of sexual spontaneity although told through humour, was quite stark for men. Some knowledge deficits about nerve sparing and its consequences was evident also. Discussing difficulties with erectile dysfunction are not topics discussed in a public sphere. For men, health is a private affair and not something that is routinely discussed by men. However, it is of note that men sought support from other men to assist their decisions to avail or not of penile rehabilitation. Ongoing challenges were evident at 6–9 months with some men describing little improvement from 3 months signaling a real need for targeted nursing intervention and assistance, especially in relation to understanding the process of penile rehabilitation. Strong relationships with partners seemed to assist in men’s sense making. Men considered intimate relationships as contributing towards their health and well-being (Robertson, 2007) and were grateful for the understanding from their female partner.

## 5 Limitations

This study is limited by virtue of the fact that it was undertaken in one tertiary hospital in Ireland during the Covid-19 pandemic. Whilst it would have been helpful to expand the study to other contexts, financial constraints prevented this from occurring.

## 6 Conclusions

Prostate cancer resulting in a radical prostatectomy has significant physical and psychological implications for masculine identity including sexual function, urinary continence, as well as changes to work, social, and intimate lives (Wennick et al., 2017). This study has highlighted the need to create spaces for men to discuss their thoughts and feelings as they evolve during and following radical prostatectomy. Healthcare professionals (in particular nurses) need to provide information that is specific and targeted which acknowledges men’s identity positioning and narrative framing which can evolve over time. Importantly men’s narrative structures can serve as a conduit to assist in meaningful understandings between nurses and patients to potentiate healthcare communication and foster a speedy recovery. The communication strategies and narratives utilised by men in this study emphasise the importance of adapting how and what nurses and healthcare professionals communicate throughout the illness and recovery journey.

## Funding

This project was funded through a Service Improvement Innovation Funding Grant (2018 ref: SI36/2018) Nursing and Midwifery Practice Development Unit, Dublin North, Ireland.

## CRediT authorship contribution statement

**Melissa Corbally:** Conceptualization, Data curation, Formal analysis, Funding acquisition, Investigation, Methodology, Project administration, Resources, Software, Supervision, Validation, Visualization, Writing – original draft, Writing – review & editing. **Catherine McGarvey:** Conceptualization, Data curation, Formal analysis, Funding acquisition, Investigation, Methodology, Project administration, Resources, Software, Validation, Visualization, Writing – original draft, Writing – review & editing. **Carla O’ Neill:** Investigation, Software, Supervision, Validation, Visualization, Writing – original draft, Writing – review & editing. **Barry Kestell:** Formal analysis, Investigation, Project administration, Software, Validation, Visualization, Writing – original draft.

## Declaration of competing interest

The authors declare the following financial interests/personal relationships which may be considered as potential competing interests:

Melissa Corbally reports financial support was provided by Nursing and Midwifery Practice Development Unit Dublin North, Ireland. If there are other authors, they declare that they have no known competing financial interests or personal relationships that could have appeared to influence the work reported in this paper.

## Appendix Supplementary materials

Image, application 1
