# Supplementary material for: Men’s accounts of erectile function over time following radical prostatectomy: A narrative study
Source: Int J Nurs Stud Adv. 2026 Jun 2;11:100574. doi: 10.1016/j.ijnsa.2026.100574 (PMC13293649; doi:10.1016/j.ijnsa.2026.100574)
Supplement: Supplementary file 1 [file mmc1.docx]

Table 1: Overview of participant group

| **Pseudonym** | **age** | **Gender** | **Pre op ED** | **Ethnicity** | **Method** | **Gleason grade** | **Nerve Spare** | **Final histology** | **Interview 1** | **Interview 2** | **Interview 3** | **In relationship** | **Previous treatment** |
| --- | --- | --- | --- | --- | --- | --- | --- | --- | --- | --- | --- | --- | --- |
| John | 64 | AMAB^[[1]](#footnote-2)^ | N | African | Rob | 3+4 | Non nerve spare | Grade group 2 pT3a | X | X | X | Y | AS |
| Nathan | 56 | AMAB | N | white Irish | Rob | 4+3 | Bilateral nerve spare | Grade Group 2 3+4 pT2 | X | X | X | Y | RXT opinion |
| Gavin | 49 | AMAB | N | white Irish | Rob | 3+4 | Unilateral right nerve spare | Grade group 2 3+4 pT2c | X | X | X | Y | AS |
| Evan | 62 | AMAB | Y | white Irish | Open | 4+4 | Non nerve spare | Grade group 3 pT3b 4+3 | X | X | X | Y | RXT opinion declined |
| Nicholas | 66 | AMAB | N | white Irish | Open | 3+4 | Non nerve spare | Grade group 2 pT2c | X | X | X | Y | AS, RXT opinion |
| Paul | 52 | AMAB | y | white Irish | Rob | 4+3 | Bilateral nerve spare | Grade group 3 4+3 pT2nx | X | X | X | Y | RXT opinion |
| James | 58 | AMAB | N | white Irish | Rob | 3+4 | Non nerve spare | Grade group 3 4+3 pT2nx | X | X | X | N | rXT not an option UR |
| Alexander | 67 | AMAB | Y | African | Rob | 3+4 | Non nerve spare | Grade group 2 pT2c | X | X |  | Y | No |
| Henry | 48 | AMAB | N | white Irish | Rob | 3+4 | Bilateral nerve spare | Grade group 2 pT2c | X |  | X | N | No |
| Glen | 60 | AMAB | N | white Irish | Rob | 3+3 | Non nerve spare | Grade group 2 pT2c | X |  |  | Y | No |
| Charles | 52 | AMAB | N | white Irish | Rob | 3+4 | Nerve sparing procedure | Grade group 2 pT2c | X |  |  | Y | No |
| William | 72 | AMAB | N | white Irish | Rob | 3+4 | Non nerve sparing | Grade group 2 pT2 | X |  |  | Y | DXT opinion |
| Joseph | 67 | AMAB | N | white Irish | Rob | 4+3 | Non nerve sparing | Grade group 3 PT3a 4+3 | X |  |  | Y | AS |
| Roger | 69 | AMAB | N | white Irish | Open | 3+4 | Non nerve sparing | Grade group 2 pT3a |  | X |  | N | No |
| Simon | 71 | AMAB | N | white Irish | Open | 4+3 | Non nerve sparing | Grade group 2 pT3a 3+4 |  | X |  | Y | DXT opinion |
| Nigel | 48 | AMAB | N | white Irish | Open | 3+4 | Unilateral nerve spare difficult | Grade group 1 pT2a |  |  | X | N | No |
| Colin | 69 | AMAB | Y | white Irish | Open | 3+4 | Non nerve sparing | Grade group 2 pT3a |  |  | X | Y | DXT opinion |
| Edward | 52 | AMAB | N | African | Open | 4+3 | unilateral nerve spare | Grade group 3 pT2a |  |  | X |  | DXT opinion |

1. Assigned Male at Birth [↑](#footnote-ref-2)
